# Isotopic composition of the eastern gray whale epidermis indicates contribution of prey outside Arctic feeding grounds

**DOI:** 10.1038/s41598-022-10780-1

**Published:** 2022-04-29

**Authors:** Michelle Gelippi, Javier Caraveo-Patiño, Marco F. W. Gauger, Brian N. Popp, Simone Panigada, Rocío Marcín-Medina

**Affiliations:** 1 Centro de Investigaciones Biológicas del Noroeste S.C., La Paz, B.C.S. México; 2 Department of Earth Sciences, University of Hawaii at Manoa, Honolulu, HI USA; 3 Tethys Research Institute, Milan, Italy; 4 Asociación de Investigación y Conservación de Mamíferos Marinos y su Hábitat A.C., La Paz, B.C.S. México

**Keywords:** Ocean sciences, Marine biology, Ecology, Stable isotope analysis

## Abstract

Eastern gray whales’ distribution range and plasticity in feeding behavior complicates the understanding of critical life-history such as pregnancy and lactation. Our goal was to determine if females who experienced gestation, gave birth, and lactated their calves, assimilated a high proportion of benthic amphipods from the Bering Sea, which are considered the species’ main prey. We used Bayesian stable isotope mixing models to estimate the probability of contribution of food items sampled along the species’ distributional range, using isotopic data on amphipods from the Bering Sea, mysids from Vancouver Island, and amphipods and polychaetes from Ojo de Liebre Lagoon. We sampled epidermal tissue from lactating females (n = 25) and calves (n = 34) and analyzed their carbon and nitrogen isotopic composition. Model outcome indicated that benthic amphipods from the Bering Sea were not the primary food for the eastern gray whale. Each mother performed a different feeding strategy, and prey from Vancouver Island were generally as important as that from the Bering Sea. Moreover, model results indicate a constant use of Ojo de Liebre Lagoon as a feeding ground. Our results appear to agree with previous studies that report continuous feeding by females to satisfy certain physiological requirements (e.g., fatty acids omega-6) during migration and breeding time. Future investigations of the isotopic composition of all those prey items that could be assimilated by the eastern gray whale emerge as critical. Both historical and recent information, that would provide insights in the species feeding ecology under past and present environmental conditions, should be considered as equally important to establish conservation and management plans.

## Introduction

Availability of food items are expected to influence whale population abundance, timing of reproduction and general reproductive output^1,2^. In the last two decades, the eastern gray whale (*Eschrichtius robustus*) population suffered unusual mortality events in 1999–2001^3–5^ and 2019–2020^6,7^, during which stranding events increased along their migratory route. Many individuals also had poor body condition, the number of recruited calves decreased, and the number of the reported feeding events outside the common feeding grounds increased. Variations in the abundance and/or availability of prey in the Bering and Chukchi Seas, the primary and secondary feeding grounds^8–10^, are normally suggested as that main cause in these phenomena.

Like most baleen whales, the gray whale is assumed to be a capital breeder, thus, costs of reproduction should be covered by using energetic and nutritive reserves stored in its lipid-reach hypodermis prior to breeding^8,11^. For this reason, trade-offs between energy accumulation (predation and metabolism) and energy consumption (food intake) are expected to be achieved in feeding grounds. For gray whales this is the Bering and Chukchi Sea, where prey availability is high during summer months (June–September)^97^. These areas host benthic communities dominated by amphipods, particularly of *Ampelisca macrocephala*, that is considered to be the main food source for the species^5,10,12^. After feeding between May and October, gray whales begin to fast and migrate south to the breeding lagoons of Baja California, in Mexico (October to January), where conception occurs between December to March. Newly pregnant females migrate back to higher latitudes (March to April) to replenish their energetic supply, and when the feeding season is over, they migrate back to the breeding grounds during the last stage of gestation (October to December). Birth and calve lactation take place from January to April, after which calves are ready to undergo their first northbound migration to the feeding areas, normally from April to June. For this scenario, fetus development, as well as timing and success of calf birth and survival, are considered directly related to the success of the pregnant mother’s feeding season^8^.

Eastern gray whale feeding ecology differs from other baleen whales because animals can use several feeding techniques, depending on different habitats and food availability^13–15^. Stomach content analysis of gray whales hunted in different parts of the Bering Sea, for example, revealed the ingestion of more than 19 genera of invertebrates, comprising amphipods, polychaetes, decapods, isopods, sponges, hydrozoas etc.^16^. Swarming species, such as cumaceans, mysids, krill, shrimps, mobile amphipods and shoal of sardines and anchovies, can also be part of whales diet^17^. Particularly in tertiary feeding grounds, as the west coast of Vancouver Island, British Columbia (Canada), and the coasts of Oregon and northern California^18–22^, where a subgroup of eastern gray whales can be found^19^, feeding may switch between planktonic (as mysids and porcelain crab larvae) and benthonic (as amphipods) food items, based on prey abundance and size^15,23^. Furthermore, visual^12,16,24,25^ and molecular evidence^12,26^, suggest that the gray whale can also feed in breeding areas. These data show how important is to understand which environmental and biological factors may influence the species reproductive outcomes, given their vast distributional range and feeding plasticity.

Direct observation of events that can influence life-history events, such as pregnancy and lactation, of marine highly mobile animals is challenging^27^. The carbon (δ^13^C) and nitrogen (δ^15^N) stable isotope analysis of whales’ epidermal structural layers is a promising tool that can be used to indirectly investigate diet during cryptic life stages^28–31^. Carbon and nitrogen isotope composition of marine primary producers differ among environments in predictable ways related to water temperature and nutrient sources that vary along latitudinal gradients^32^. δ^13^C values are useful to investigate animal movements and determine dietary carbon sources, and δ^15^N values can indicate trophic connections and diet preferences^33,34^. In each local food web, δ^13^C and δ^15^N values of basal nutrients are reflected throughout trophic levels, and increase by 0.5–3.0‰ and 2.0–5.0‰, respectively, at each trophic step, depending on which species and tissues are analyzed^35,36^. Precise estimates of tissue-to-diet isotope discrimination can be obtained from controlled feeding experiments, which are nearly impossible to perform with large free-ranging animals, such as the gray whale. Among all cetaceans, δ^13^C and δ^15^N mean trophic discrimination factors for epidermis were estimated experimentally only for the bottlenose dolphin (*Tursiops truncatus*)^37,38^. Gimenez et al*.* (2016)^37^, which performed longer experimental trials than Browning et al*.*^38^, reported trophic discrimination factors of 1.0 ± 0.4‰ for δ^13^C values and 1.74 ± 0.55‰ for δ^15^N values. On the other hand, trophic discrimination factors were obtained by modelling epidermis δ^15^N values of free ranging blue whales (*Balaenoptera musculus*)^28^, and epidermal δ^13^C and δ^15^N values of dead caught fin whales (*Balaenoptera physalus*)^39^. Specifically, for the blue whale a δ^15^N trophic discrimination factor was estimated to be 1.8 ± 0.3‰^28^, and for the fin whale δ^13^C and δ^15^N discrimination factors of 1.28‰ and 2.82‰, respectively^39^. Blue whale trophic discrimination factors were estimated from individuals that were most probably in steady state with their sources^28^, while it is not known if fin whales epidermis were in steady state with their main prey^39^.

Epidermis is different from metabolically active tissues (as plasma and muscles) in that it grows continuously and provides an ~ 70 day archive of isotopic compositions in its three layers, the innermost “stratum basale”(SB), the intermediate “stratum spinosum” (SS) and the outermost “stratum corneum” (SC)^40,41^. Therefore δ^13^C and δ^15^N values in each epidermal layer can reflect those of diet integrated over specific periods of tissue growth^28–31^. When new cells are formed in the SB, their δ^13^C and δ^15^N values reflect that of blood stream components^42^. After ~ 10–20 days, these cells leave the SB and enter first the SS, where they move along for ~ 20–50 days, and then the SC, where they conclude their life-span after ~ 70 days^43,44^. Recently, temporal changes in feeding preferences and seasonal and regional movements of different species of baleen whales were reflected in epidermal inter-layer δ^13^C and δ^15^N variability in blue^28,29^, humpback, sperm and fin whale^30^. Differences among epidermal layer isotope values are reported for the gray whale^31^. Specifically, variations in inter-layer isotope values appear to exist in lactating mothers and calves, possibly due to the effect of physiological/dietary transition from gestation (placental blood) to lactation (maternal milk). Based on this evidence, it appears possible to investigate the origin of energetic sources used during pregnancy and lactation by analyzing epidermal layer δ^13^C and δ^15^N values.

In this study, we used stable isotope mixing models to assess the probable contribution of resources from primary and tertiary feeding grounds to the δ^13^C and δ^15^N values of the newest (SB) and oldest (SC) epidermal layers of gray whale calves and lactating females, sampled during three years with different calf recruitments. Based on the assumption that gray whale reproductive output should be positively correlated to the availability of its main prey (*Ampelisca macrocephala*) in the Bering Sea^8,11,45^, we expected to verify three main hypothesis. First, all females that accomplished gestation, gave birth and successfully lactated their calves, assimilated in their epidermis high proportions of benthic amphipods from the Bering Sea. Second, resources from tertiary feeding grounds would not influence epidermal δ^13^C and δ^15^N values of lactating females. In these areas, feeding is indeed considered only sporadic and transitory^8,15^. Third, estimates of resource partitioning would not differ based on which epidermal layer is analyzed; because all gray whales undergo fasting during the last stage of pregnancy, and through the whole lactation phase^4,8,46^.

## Methods

### Permits, ethic statement and approval

Skin biopsies were obtained in accordance with the relevant guidelines and regulations imposed by the Mexican Secretariat of Environment and Natural Resources (SEMARNAT) and under sampling permits n: SGPA/DGVS/0937, SGPA/DGVS/011543/17, SGPA/DGVS/010876/18 and SGPA/DGVS/12644/19, released from the same Mexican Institution. The research permits also included the necessary ethical approval in terms of sample collection, analysis and use for scientific studies.

### Sample collection

**Figure 1:**
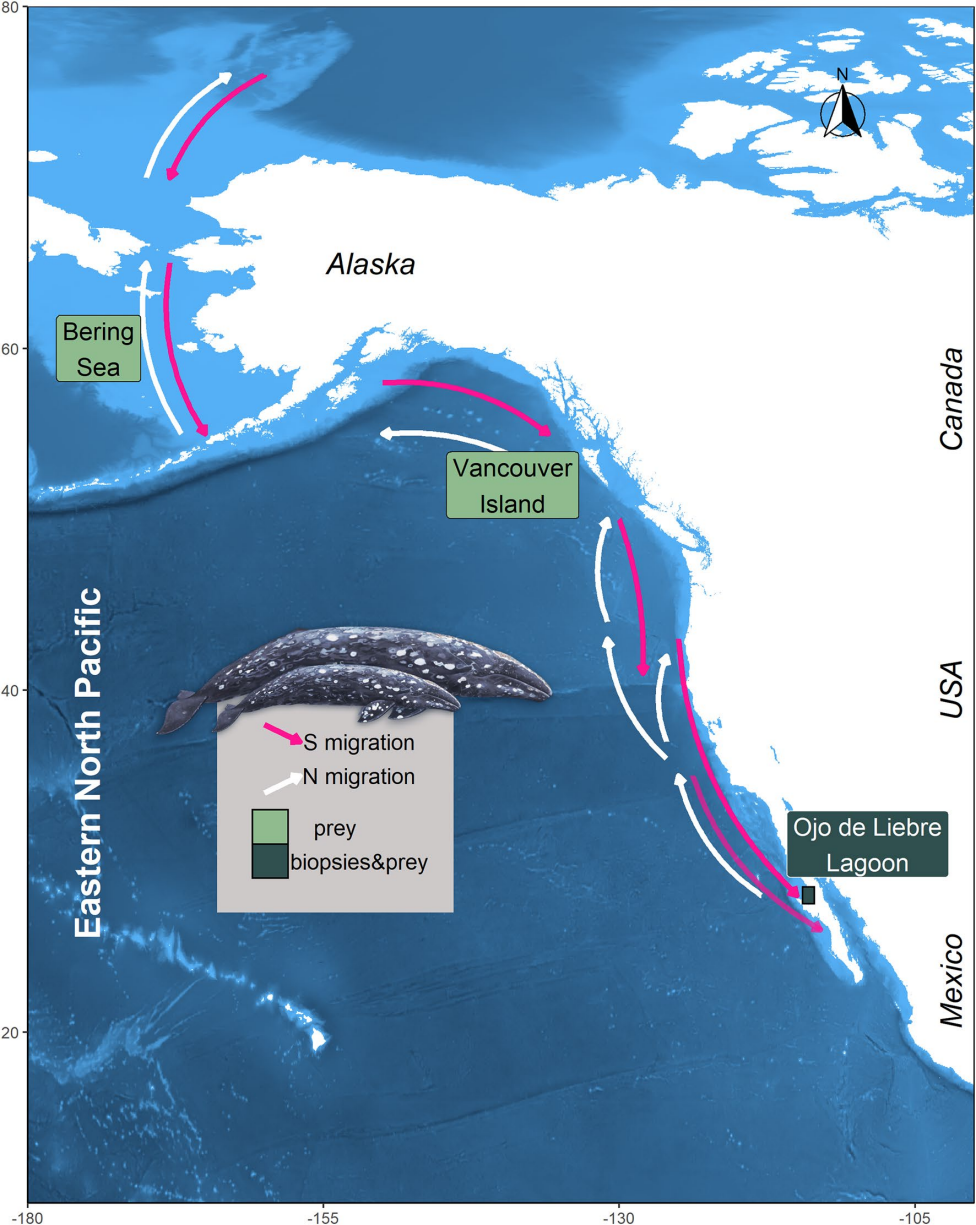
Arrows represent the eastern gray whale migration route between northern feeding areas and southern breeding lagoons. Possible prey were collected in the Bering Sea, Vancouver Island and Ojo de Liebre Lagoon. Gray whale mothers and calves’ skin samples were collected in Ojo de Liebre Lagoon. Map was created using the software R, version 3.6.0, library “marmap”.

Epidermis samples of free-ranging gray whale calves and lactating mothers were collected in the breeding ground Ojo de Liebre Lagoon (Latitude: 27.75; Longitude: − 114.25) (Fig. 1), Baja California Sur, Mexico, in February 2011 and 2018, January 2019, and March 2019. Samples were collected as described in Gelippi *et al.*^31^. In brief, focal sampling methodologies^47^ and photoidentification techniques^48,49^ were applied to all specimens except those collected in 2011. Biopsy samples were collected either with a 5 m long stainless steel pole equipped with a modified stainless steel biopsy point^50^ (samples from 2011, 2018 and calves from January 2019), or with a crossbow armed with stainless-steel biopsy darts’ tip^51^ (mothers from January 2019 and all mothers and calves from March 2019). Independent of sampling methodology, epidermis was always taken from the dorsal portion of animals, between the dorsal fin and the fluke. Biopsies were wrapped in aluminum foil, placed inside sterile plastic bags, and stored on ice until land was reached (usually less than 5 h). Thereafter, samples were stored in liquid nitrogen and then frozen at − 80 °C. Between each biopsy event, tip and dart were cleaned, sterilized, and stored in alcohol.

Prey samples from gray whale primary feeding grounds in the Bering Sea and from west of Vancouver Island (Fig. 1), which is considered a tertiary alimentation area, were available from previous scientific expeditions. Specifically, benthonic amphipods ampeliscid (*Ampelisca macrocephala*) were collected from sixty stations located longitudinally across the whole Bering Sea, by the scientific cruise Alpha-Helix of the University of Alaska (Fairbanks), during gray whale feeding season of summer 2002^52^. In this work five of those sixty stations were selected, and corresponding amphipods were analyzed. Stations were chosen based on an east–west gradient, from inshore to offshore, to assure that possible prey variability associated with different sampling locations would be considered in the analysis. Prey collections near Vancouver Island took place where gray whales were observed to feed in the water column during summer 2002. Samples from four different locations were composed of pelagic mysids (family Mysidae), and all those invertebrates were analyzed. Based on previous evidence that suggested gray whale feeding activity on amphipods living in seagrass beds inside the breeding lagoons^12,24,25^, during winter 2018 benthic samples (n = 5) were taken with an Ekman dredge (6′ × 6′ × 6′) from seagrass mats areas in Ojo de Liebre Lagoon (Fig. 1). In the same time frame, more benthic samples (n = 2) were dredged where whales were seen feeding (i.e., repeated diving events, presence of mud in the water column, outflow of mud and water from gray whales’ mouth, presence of feces). After collection, benthic samples were washed with seawater through a 0.5 mm screen and stored on ice until land was reached. Organisms were then sorted manually, pooled together by order to average local variation, and finally frozen at − 80 °C.

### Stable isotope analysis

All samples were processed at Centro de Investigaciones Biológicas del Noroeste, La Paz, Baja California Sur, Mexico. Frozen biopsies were cut with a sterile scalpel to separate the epidermis from the dermis and the hypodermis. Successively, each epidermis was subsampled at its outermost part, at the half of its length and close to the dermis. Those 3 subsamples were considered to represent the 3 structural layers of the epidermis, respectively, SC, SS and SB. Total lipids were extracted^53^ from both biopsies and prey samples (2:1 chloroform–methanol, 24 h) prior to freeze-drying. Stable isotope analysis of whole prey and of biopsies collected in 2011 and 2018 was performed at the SOEST Stable Isotope Biogeochemical Facility of the University of Hawai ‘i at Mānoa and biopsies collected in 2019 were analyzed at U.C. Davis Stable Isotope Facility. Samples were powdered and weighed into tin cups (Mānoa: 0.5 mg ± 0.05; Davis: 0.1 mg ± 0.02), and their C and N isotopic composition was measured using a Costech elemental combustion system (Model 4010), coupled to a Thermo-Finnigan Delta plus XP isotope ratio mass spectrometer through a Conflo IV interface (Mānoa), and with an Elementar Vario Micro Cube elemental analyzer (Elementar Analysensysteme GmbH, Hanau, Germany) interfaced to an Isoprime VisION isotope ratio mass spectrometer (Davis). Glycine and tuna muscle tissue were used as reference materials to calibrate the samples and to correct for instrument drift at Mānoa, and Alfalfa flower, bovine liver, enriched alanine, glutamic acid, and nylon 6 at Davis. All isotope values are expressed in delta (δ) notation relative to V-PDB for carbon and atmospheric nitrogen. Accuracy and precision at University of Hawai ‘i and U.C. Davis were < 0.2‰, as determined from multiple laboratory reference materials extensively calibrated using National Institute of Science and Technology reference materials and analyzed every 10 samples.

### Inference of placental blood and maternal milk δ^13^C and δ^15^N values

A previous investigation showed that changes in δ^13^C and the δ^15^N values among gray whale mother-calf epidermal layers corresponded to the transition from gestation to lactation^31^. Based on this, the isotopic composition of calves’ SC and SB should represent the effects of intra and extra uterine life, respectively, thus of the assimilation of placenta blood and maternal milk. Given that these last tissues are particularly difficult to collect from free-ranging and alive individuals, the isotopic composition of fetus and calf diet (δ_diet_) was here predicted using the equation^54^:

1 $$\delta_{epidermis} = \delta_{diet} + \Delta_{dt}$$

where $\delta_{epidermis}$ is the δ^13^C and the δ^15^N value of a specific epidermal layers and $\Delta_{dt}$ is the trophic discrimination factor (TDF) between epidermis and diet. If it is true that nutrient composition of fetus/calf diet is strictly related to that of its mother, since the female catabolizes its own tissues to nourish its young^55^, than the fetus/calf should feed at one trophic level higher than its mother^56^. Since gray whale epidermis-prey TDF is unknown, δ^13^C_diet_ and δ^15^N_diet_ were calculated based on each calf’s SC and SB, and TDF based on experimental estimates from bottlenose dolphins’ epidermis SC (i.e., 0.93 ± 0.56 for δ^13^C and 1.74 ± 0.55 for δ^15^N)^37^.

### Statistical analysis

Statistical analysis and graphical visualizations were performed with the statistical software package R version 3.6.0 for Windows (R core Team 2017). Null hypotheses were rejected when p value was lower than 0.05.

Previous modelling of gray whale epidermal δ^13^C and δ^15^N values^31^ indicated significant differences depending on which epidermal layer was considered (SC, SS, SB), which age class (mother or calf), and on the interaction between these two factors. Moreover, a year-to-year variability was observed between mean epidermal δ^13^C and δ^15^N values, but authors did not test for it, and considered it rather as a random variable. Here, we used linear mixed effect models (“lme”) (R package “lme4”), first to corroborate that the inclusion of isotope data from individuals collected in March 2019 would still highlight the isotope variation found by Gelippi et al*.*^31^. Second, we set the variables “year” and “month” as fixed effects, to test if lactating mothers and calves’ epidermal layer δ^13^C and δ^15^N values would vary significantly between sampling periods. Models’ parametrization included first random and then fixed variables (forward model selection). The most parsimonious model was selected based on the lowest AICc value and the highest AICc weight (“AICcwt”). Models’ random effects accounted for repeated measures of individual’s epidermal layer isotopic values (ID_layers_), and the mother-calf pair isotopic link (ID_mcp_) due to unilateral food transfer during gestation and lactation. Values were allowed to vary within the boundaries of an individual ID and within the boundaries of mother-calf pairs IDs (1 | ID_mcp_/ID_layers_^57^). Fixed effects were “layers” (SC/SS/SB), “age” (mother/calf), the interaction between “layers” and “age” (“layers*age), and year and month of sampling (YM: February 2011/February 2018/January 2019/March 2019). Variables were tested for multicollinearity and residuals plots were inspected visually to detect possible deviation from homoscedasticity and normality (“performance” package). A deviation from normality with less than 6 atypical values resulted in the exclusion of these data points. On the contrary, with more than 6 atypical values the most parsimonious model was parameterized with robust statistic (“robustlmm” R package). Estimated marginal means (EMM, “emmeans” R package) were computed and p-values adjusted for multiple comparison by Holm. This post-hoc analysis was performed to estimate layer specific interannual differences in mothers and in calves.

### Estimates of prey contribution

Bayesian stable isotope mixing models^58^ were used to calculate the relative contribution of different possible prey to the isotopic composition of calves and lactating females epidermis. Bayesian models are useful because they allow uncertainty related to inter-individual differences in isotopic composition of both consumers and prey samples to be considered based on uncertainty in measured isotope values and trophic discrimination factors^59–61^. Isotope mixing models were performed using the “simmr” R package, which is the latest upgrade of the SIAR package^62^. Models were fitted with (1) mean δ^13^C and δ^15^N values and corresponding standard errors of probable food sources collected in the Bering Sea, Vancouver Island and Ojo de Liebre Lagoon; (2) δ^13^C and δ^15^N values of females SC, females SB, estimated placental blood (from calves SC) and estimated maternal milk (from calves SB); (3) δ^13^C and δ^15^N TDFs and their associated standard errors (i.e., 0.93 ± 0.56 and of 1.74 ± 0.55^37^).

First stable isotope mixing models, here defined as *a-priori* models, were used to evaluated the probability of contribution of different prey to the isotopic mixture of female’s SC. Cetaceans in general^28,43^, and gray whales in particular^31^, can integrate in their SC isotopic inputs of up to 70 days, a time that represents northern feeding or southbound migration, based on sampling time. Because of this, it was assumed that model estimates for females’ SC would indicate a higher *a-priori* probability of contribution for Bering Sea amphipods, thought to be the main source of carbon and nitrogen for the eastern gray whale population^8^. Due to the lack of previous information, it was unknown if and how much Ojo de Liebre prey could contribute to the isotopic composition of gray whale epidermis. For this reason, *a-priori* model outcomes indicated which Ojo de Liebre source had the higher probability of contributing to each female’s SC isotopic mixture. Based on these results, a second stable isotope mixing model, here defined as *a-posteriori* models, was used to test the probability of contribution of Bering Sea amphipods, Vancouver Island mysids and of the selected Ojo de Liebre Lagoon prey, to each female’s SC. These a*-posteriori* models were also used to calculate the probable isotopic contribution of selected sources to females’ SB, inferred from placental blood (i.e., calves’ SC) and maternal milk (i.e., calves SB). *A-posteriori* models were run for mother-calf pairs using the same prey selection (defined by *a-priori* outcomes). In the case of alone calves, on the other hand, *a-posteriori* models were fitted with the source selection identified as most relevant for the highest number of females sampled in the corresponding month and year. *A-posteriori* models’ results were visualized through ternary plots^63^ (“ggtern” R package). These plots consist of equilateral triangles, where each vertex represents the percent contribution (0–100%) of one of the three sources, and each sample adds up to 100%. Distinct colorations indicate if individual samples had higher probability to have assimilated amphipods or polychaetes from Ojo de Liebre Lagoon.

General Linear Mixed Effect models (“GLME”) were used (“glmmTMB” R package) to test differences in sources contributions, under the assumption of a beta error distribution (0–100%). An independent GLME was used to determine the contribution from each source, and model results were then merged and visualized together. We used this approach to allow the most parsimonious model reflected area-specific differences. A unique model, on the other hand, might have homogenized these differences, or estimate artifacts. Each GLME was parametrized as the “lme” presented above. Estimated marginal means (EMM, “emmeans” package) were computed and p-values adjusted for multiple comparison by Holm. This post-hoc analysis was performed to estimate differences in the percentages of sources contribution among individual layers during different months and years.

## Results

### Gray whale epidermal layers isotopic patterns

**Figure 2:**
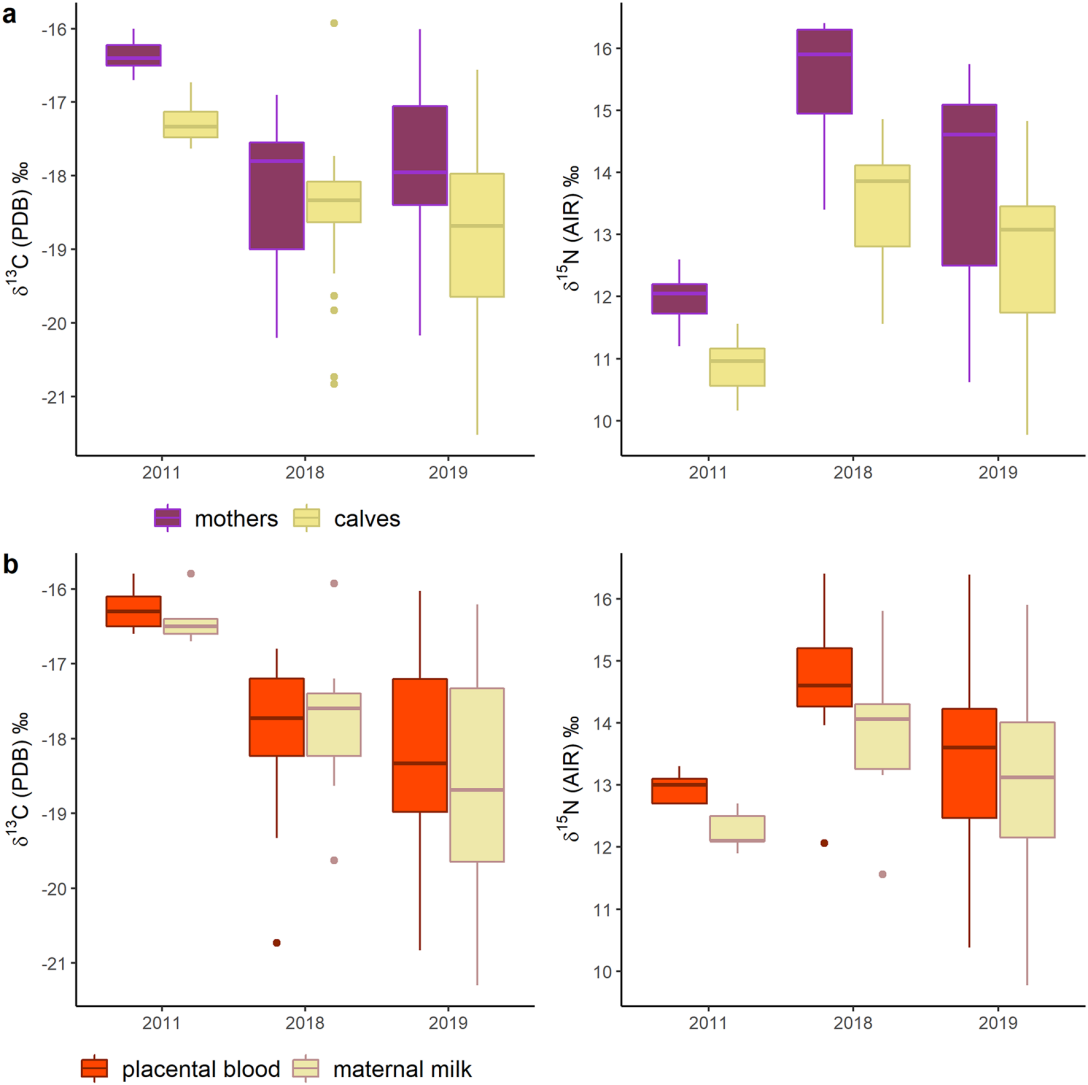
Boxplots of the mean δ^13^C and δ^15^N values of gray whale mothers and calves’ epidermis collected in 2011, 2018 and 2019 (**a**), and mean estimated δ^13^C and δ^15^N values of placental blood and maternal milk (**b**) using Eq. (1).

Epidermal δ^13^C and δ^15^N values for 20 eastern gray whale mother-calf pairs, plus 5 lactating females and 14 calves sampled alone, ranged from − 20.2 to − 16.0 and from 10.3 to 16.4, respectively (Fig. 2a). Mean estimated δ^13^C and δ^15^N values for placental blood ranged from − 20.8 to − 15.8 and from 10.4 to 16.4, respectively, and those of maternal milk from − 21.3 to − 15.8 and from 9.8 to 15.9 (Fig. 2b). The most parsimonious model for carbon indicated that δ^13^C values varied significantly in females and in calves due to the factors “layers”, “age” and “year and month” (AICc = 377.71, AICcwt = 0.29, supplementary Table S1). On the other hand, for δ^15^N values, the best fitted model indicated that “layers”, “age”, “layer*age” and “year and month” significantly influenced observed variation (AICc = 287.74, AICcwt = 0.46, supplementary Table S2). In both δ^13^C and δ^15^N models, residuals deviated from the normality assumption, and more than 6 values were atypical (Supplementary Figs. S3 and S4). The best models were run using robust statistics as to lower the importance of atypical values (weighting), and to estimate the unbiased coefficients (Supplementary Tables S5 and S6). 95% confidence intervals were higher for estimated δ^13^C and δ^15^N patterns for both females and calves. Post-hoc analysis indicated significant differences among mother-calf pairs δ^13^C and δ^15^N values within years of sampling. When year-to-year differences were considered, epidermal δ^13^C values of females and calves varied between 2011 and 2019 (*p* < 0.01, Supplementary Table S7). δ^15^N values showed significant differences between all sampling years for mothers (2011–2018 & 2011–2019: *p* < 0.001, 2018–2019: *p* < 0.05, Supplementary Table S8), while nitrogen isotope patterns of calves differed significantly in only animals sampled in 2011 (2011–2018: *p* < 0.001, 2011–2019: *p* < 0.05).

### Estimates of source contribution to the eastern gray whale epidermal isotopic mixtures

Benthic samples of seagrass mats collected in Ojo de Liebre Lagoon contained only small (< 2 cm) amphipods, while big (> 10 cm) polychaetes were found in areas where gray whale feeding behavior was observed. Mean δ^13^C and δ^15^N values were − 15.5 ± 0.3 and 15.5 ± 0.2 for amphipods, and − 15.0 ± 1.1 and 7.1 ± 1.6 for polychaetes. Carbon and nitrogen isotope mean values for Bering Sea amphipods and for Vancouver Island mysids were − 20.3 ± 1.0 and 9.3 ± 1.0, and − 16.8 ± 3.8 and 11.1 ± 1.0, respectively.

**Figure 3:**
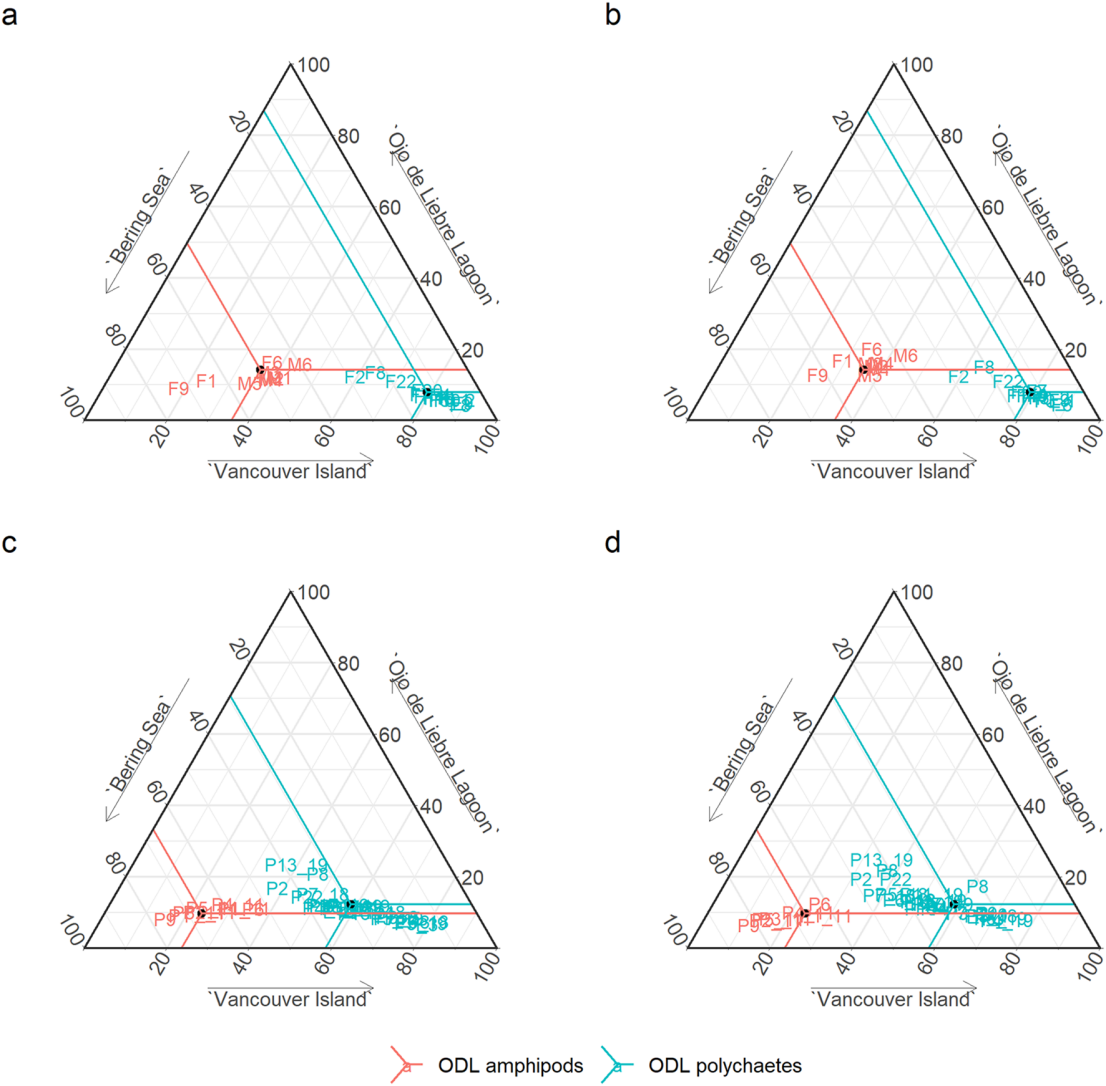
Ternary plots, representing the estimated percentages of contribution of the three considered sources to the isotopic mixture of gray whale tissues. Points coloration pattern shows which source between amphipods (red color) and polychaetes (light-blue color) from the breeding lagoon (ODL = Ojo de Liebre Lagoon) had a higher probability to influence the isotopic mixture of mothers’ SC (**a**), mothers’ SB (**b**), estimated placental blood (i.e., calves’ SC, (**c**) and estimated maternal milk (i.e., calves’ SB, **d**).

*A-priori* models indicated that the probability of contribution of Bering Sea amphipods was not the highest in all females’ SC (Supplementary Table S9). Patterns of contribution were individual-specific. To evaluate and understand the effects of year-to-year changes on a broader scale, the probability of contribution of all possible prey were evaluated. Results for lactating females showed high variability in the contribution patterns of different food sources in SC and SB (Fig. 3a,b). Bering Sea amphipods were estimated to have contributed most to the carbon and nitrogen isotope composition of female’s epidermis in only 13% of all cases. In contrast, Vancouver Island mysids contributed 60% of all mother’s epidermis and the most probable estimated scenario for the remaining 26% was that both Bering Sea and Vancouver Island carbon and nitrogen sources were assimilated in equal proportions in their tissues. Despite the low predicted contribution of Ojo de Liebre Lagoon sources (< 20%), outcomes suggest that when amphipods were consumed in the Arctic, they were also consumed in the breeding ground. On the contrary, when Vancouver Island mysids had the highest probability of contributing to females’ isotopic mixture, polychaetes were preferred in Ojo de Liebre Lagoon (Fig. 3a,b). Concerning model outcomes for estimated placental blood and maternal milk (Fig. 3c,d), we observed patterns comparable to those of mothers’ epidermis (Fig. 3a,b). Precisely, Bering Sea amphipods and Vancouver Island mysids had the highest estimated contribution to placental blood and maternal milk with a probability of 22% and 64%, respectively (Fig. 3). Ojo de Liebre prey contribution was predicted to vary among individuals, and, in some cases, it was > 20%.

**Figure 4:**
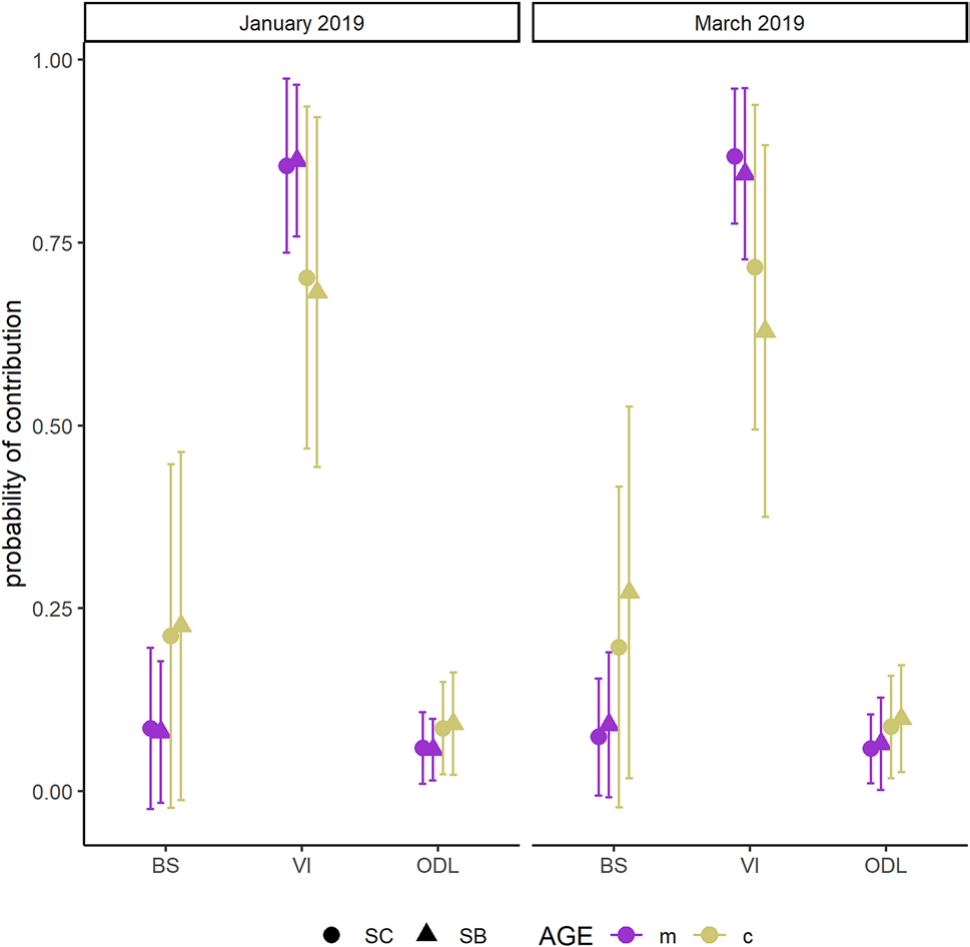
Predicted percentages of contribution of the three sources (BS = Bering Sea, VI = Vancouver Island, ODL = Ojo de Liebre Lagoon) to the isotopic mixture of the mother-calf pair collected and recollected in January 2019 and March 2019. For calf, SC and SB are representing estimated placental blood and maternal milk.

The 28th of January and the 15th of March 2019 the same mother-calf pair was recaptured and resampled. The corresponding *a-posteriori* models results are shown in Fig. 4. The estimated probabilities of contribution changed little among sampling months, and Vancouver Island mysids were the highest probable contributors to the isotopic mixture of mother’s epidermis, estimated placental blood, and estimated milk. The uncertainty (i.e., standard error) associated to the estimated percentages of contribution for Bering Sea and Vancouver Island sources to placental blood and maternal milk was higher than for mother’s epidermis. On the other hand, the probable contribution of Ojo de Liebre Lagoon polychaetes (indicated by *a-priori* models as the prey with the highest probability of contribution in the breeding lagoon, Supplementary Table S9, code F3_1/F3_2) was similar for mothers’ epidermis, estimated placental blood and estimated milk.

**Figure 5:**
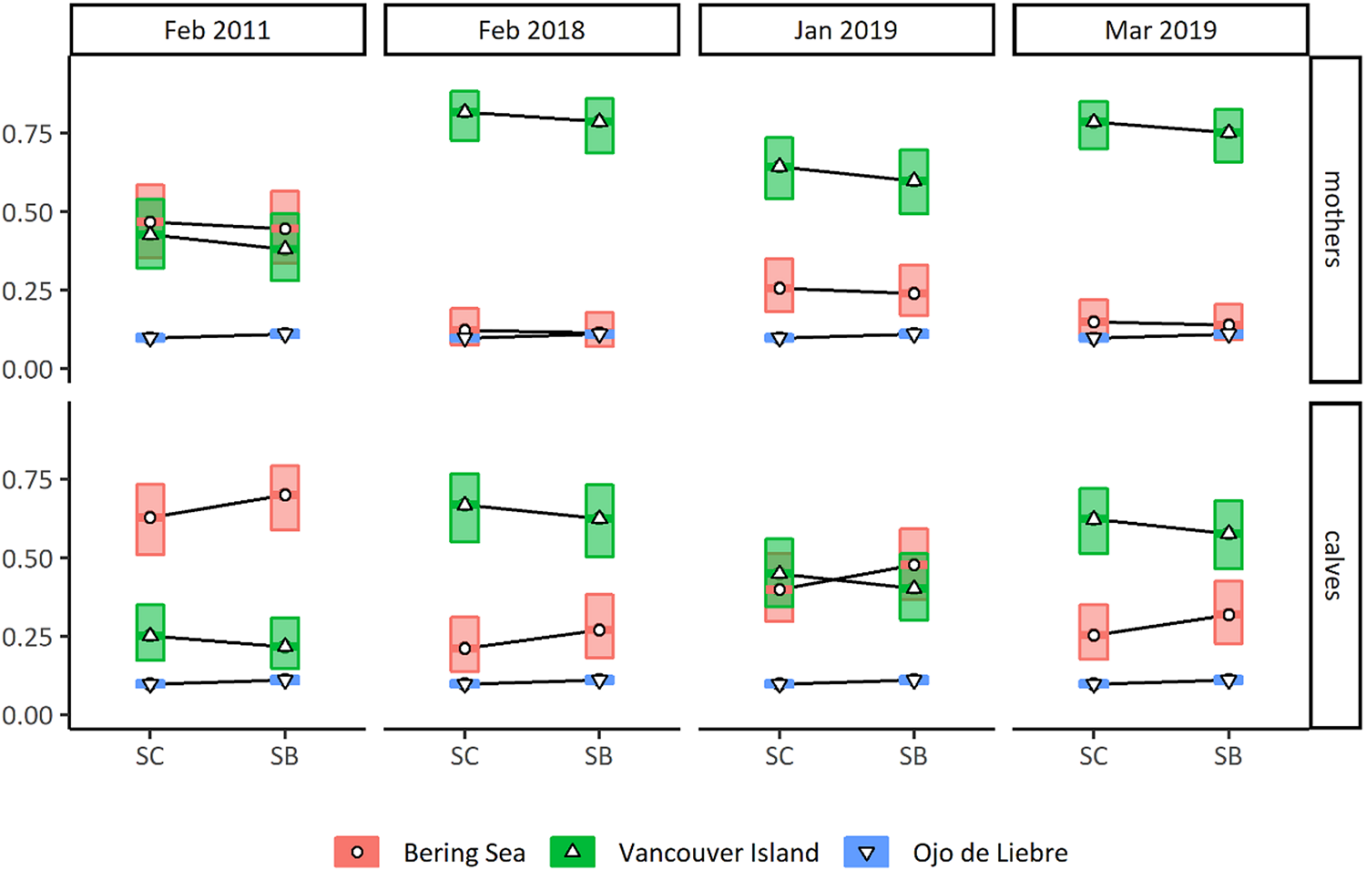
Top-selected general linear mixed effect model for all sources contribution patterns to the isotopic mixture of mothers’ epidermis, placental blood (i.e., calves SC) and maternal milk (i.e., calves SB). Boxes indicate the upper and lower confidence levels of the estimated marginal means.

The most parsimonious GLME confirmed that sources were contributing to the isotopic mixtures following variable patterns (Fig. 5, Supplementary Tables S10-S12). The model indicated that factors responsible for differences between contributions of Bering Sea and Vancouver Island prey were “layers”, “age” and “year and month”, plus the interaction “layers*age” for Bering Sea prey only. GLME outcomes predicted that the contribution of both Bering Sea and Vancouver Island sources would be higher in mothers’ SC than in their SB. Furthermore, models predicted that Bering Sea prey contribution would be higher in estimated maternal milks than in estimated placental bloods. Only Vancouver Island sources appeared to contribute more to estimated placental bloods than to estimated maternal milks. The best fitted GLME indicated that only the factor “layers” was determined as a significant difference for Ojo de Liebre Lagoon prey contributions. No apparent problems were detected due to multicollinearity. The visual inspection of residuals revealed deviation from normality, which, however, was minor and, consequently, did not determined model rejection^64^. Post-hoc evaluation confirmed that Bering Sea (Table 1) and Vancouver Island (Table 2) prey contribution differed significantly among all groups as determined by the best fitted GLME. Moreover, the analysis indicated the probability of Ojo de Liebre Lagoon prey contribution increased significantly (p = 0.0001) between females’ SC and SB isotopic mixtures, and between those of placental blood and maternal milk.

**Table 1:** Post-hoc analysis results for general linear mixed effect model predictions for Bering Sea amphipods contribution based on “layer” (SC, SB) within each “age” (mothers; calves) and between “year and month” of sampling.

Prey location	index	Group A	Group B	Odds. ratio	SE	*df*	t.ratio	*p* value
Bering Sea	Mothers	feb_2011 SC	feb_2018 SC	6.32	2.04	86	5.70	0.00002
		feb_2011 SC	jan_2019 SC	2.55	0.75		3.19	0.12630
		feb_2011 SC	mar_2019 SC	4.99	1.50		5.36	0.00007
		feb_2018 SC	jan_2019 SC	0.40	0.13		− 2.91	0.23720
		feb_2018 SC	mar_2019 SC	0.79	0.25		− 0.74	1.00000
		jan_2019 SC	mar_2019 SC	1.96	0.56		2.33	0.89728
		feb_2011 SB	feb_2018 SB	6.32	2.04		5.70	0.00002
		feb_2011 SB	jan_2019 SB	2.55	0.75		3.19	0.12630
		feb_2011 SB	mar_2019 SB	4.99	1.50		5.36	0.00007
		feb_2018 SB	jan_2019 SB	0.40	0.13		− 2.91	0.23720
		feb_2018 SB	mar_2019 SB	0.79	0.25		− 0.74	1.00000
		jan_2019 SB	mar_2019 SB	1.96	0.56		2.33	0.89728
	Calves	feb_2011 SC	feb_2018 SC	6.32	2.04	86	5.70	0.00002
		feb_2011 SC	jan_2019 SC	2.55	0.75		3.19	0.12630
		feb_2011 SC	mar_2019 SC	4.99	1.50		5.36	0.00007
		feb_2018 SC	jan_2019 SC	0.40	0.13		− 2.91	0.23720
		feb_2018 SC	mar_2019 SC	0.79	0.25		− 0.74	1.00000
		jan_2019 SC	mar_2019 SC	1.96	0.56		2.33	0.89728
		feb_2011 SB	feb_2018 SB	6.32	2.04		5.70	0.00002
		feb_2011 SB	jan_2019 SB	2.55	0.75		3.19	0.12630
		feb_2011 SB	mar_2019 SB	4.99	1.50		5.36	0.00007
		feb_2018 SB	jan_2019 SB	0.40	0.13		− 2.91	0.23720
		feb_2018 SB	mar_2019 SB	0.79	0.25		− 0.74	1.00000
		jan_2019 SB	mar_2019 SB	1.96	0.56		2.33	0.89728

**Table 2:** Post-hoc analysis results for general linear mixed effect model predictions for Vancouver Island mysids contribution based on “layer” (SC, SB) within each “age” (mothers; calves) and between “year and month” of sampling.

Prey location	Index	Group A	Group B	Odds. ratio	SE	*df*	t. ratio	*p* value
Vancouver Island	Mothers	feb_2011 SC	feb_2018 SC	0.17	0.06	86	− 5.37	0.000067
		feb_2011 SC	jan_2019 SC	0.41	0.12		− 2.92	0.246696
		feb_2011 SC	mar_2019 SC	0.21	0.06		− 5.15	0.000159
		feb_2018 SC	jan_2019 SC	2.46	0.79		2.81	0.322270
		feb_2018 SC	mar_2019 SC	1.22	0.40		0.61	1.000000
		jan_2019 SC	mar_2019 SC	0.50	0.15		− 2.37	0.771440
		feb_2011 SB	feb_2018 SB	0.17	0.06		− 5.37	0.000067
		feb_2011 SB	jan_2019 SB	0.41	0.12		− 2.92	0.246696
		feb_2011 SB	mar_2019 SB	0.21	0.06		− 5.15	0.000159
		feb_2018 SB	jan_2019 SB	2.46	0.79		2.81	0.322270
		feb_2018 SB	mar_2019 SB	1.22	0.40		0.61	1.000000
		jan_2019 SB	mar_2019 SB	0.50	0.15		− 2.37	0.771440
	Calves	feb_2011 SC	feb_2018 SC	0.17	0.06	86	− 5.37	0.000067
		feb_2011 SC	jan_2019 SC	0.41	0.12		− 2.92	0.246696
		feb_2011 SC	mar_2019 SC	0.21	0.06		− 5.15	0.000159
		feb_2018 SC	jan_2019 SC	2.46	0.79		2.81	0.322270
		feb_2018 SC	mar_2019 SC	1.22	0.40		0.61	1.000000
		jan_2019 SC	mar_2019 SC	0.50	0.15		− 2.37	0.771440
		feb_2011 SB	feb_2018 SB	0.17	0.06		− 5.37	0.000067
		feb_2011 SB	jan_2019 SB	0.41	0.12		− 2.92	0.246696
		feb_2011 SB	mar_2019 SB	0.21	0.06		− 5.15	0.000159
		feb_2018 SB	jan_2019 SB	2.46	0.79		2.81	0.322270
		feb_2018 SB	mar_2019 SB	1.22	0.40		0.61	1.000000
		jan_2019 SB	mar_2019 SB	0.50	0.15		− 2.37	0.771440

## Discussion

Marine conservation and management plans should be established primarily based on the identification of those areas where food sources can satisfy animal’s physiological requirements of reproduction^65^. Under this perspective, we considered isotopic analysis of epidermal layers a useful tool to speculate about the eastern gray whale feeding ecology during gestation and lactation. Unexpectedly, *a-priori* and *a-posteriori* models results did not support our primary hypothesis that the highest probability of contribution to the diet of females’ is always Bering Sea amphipods. Even during a year of high numbers of recruited calves, mysids of Vancouver Island were estimated to contribute importantly to the feeding ecology of pregnant and lactating females. Furthermore, data modelling pointed out that probable contribution of prey from Ojo de Liebre Lagoon increased during the time that animals spend in the breeding lagoon. This observation was made independently from year of sampling, hence, both in periods with high and low recruitment rates. We speculate that gray whale continuous feed during reproduction. Feeding patterns varied among individuals, which appears to support previous work suggesting ecological plasticity in foraging eastern gray whales^13,15,23^. Our overall findings suggest that evaluation and understanding of year-to-year environmental changes on the eastern gray whale population should consider several large marine ecosystems, including those of the breeding lagoons.

The feeding ecology of the eastern gray whale is not commonly investigated through stable isotope analysis. Probably, because the species is classified as a high-Arctic feeder of benthic amphipod beds^15^, while foraging on other prey and in other areas is considered marginal ^158^. Consequently, tissues δ^13^C and δ^15^N values of all gray whales should be comparable among individuals. In this theoretical framework, whales tissues should reach isotopic steady-state with prey, with times that are tissue-specific^66–68^, once they move outside the main feeding grounds, because fasting is supposed to last up to 7 months^8^. Among all tissues, epidermis appears particularly useful to investigate the origin of those carbon and nitrogen sources accumulated in gray whale blubber and used during life-stages as pregnancy and lactation. Epidermal structural proteins seem to not undergo catabolism during nutritional stress^69^, a condition that can affect especially δ^15^N values^70^. Despite these considerations, however, recent results of gray whale epidermal δ^13^C and δ^15^N values do not clearly support temporal and inter-individual isotopic homogeneity during fasting. Carbon and nitrogen isotopic composition of epidermis sampled from fifteen stranded animals along the California coast, from 2000 to 2011, appeared to be comparable between individuals of different age classes and sex^71^. Conversely, epidermal δ^13^C and δ^15^N values were different in free-ranging calves and lactating females, on an inter and intra-individual base^31^. This last study, moreover, pointed out a difference in mean epidermal δ^15^N values between years of sampling, which led authors to hypothesize that the transition from gestation to lactation affected differently the nitrogen isotope composition of each female. Nevertheless, no diet estimate was carried out, consequently, it could not be excluded that individuals’ isotopic composition reflected variation in feeding ecology. Here, we remodeled those data^31^, and we added new results from mothers and calves sampled in March 2019. By setting “year” as fixed effect instead of random effect, we confirmed that epidermal δ^13^C and δ^15^N values were influenced significantly by this variable, in addition to age class (mother/calf) and epidermal layers (SC/SS/SB) (Supplementary Tables and Figs. S1-S8). Moreover, the variability of female δ^13^C and δ^15^N values in epidermal layers increased from the outermost to the innermost layer, thus, we assumed that other processes beside gestation and lactation^31^ could influence the isotopic composition of gray whale mothers.

Marine ecosystems are characterized by plankton that integrate δ^13^C and nitrate δ^15^N values that differ along a latitudinal gradient, which are then propagated through a food web^32^. During pregnancy, the eastern gray whale migrates between high and temperate latitudes (Fig. 1), thus, among ecosystems with different δ^13^C and δ^15^N values. In this work, prey isotopic ratios confirmed this latitudinal gradient, with lowest δ^13^C and δ^15^N values in Bering Sea amphipods (i.e., − 20.3 ± 1.0 and 9.3 ± 1.0), highest in Ojo de Liebre Lagoon invertebrates (i.e., amphipods: − 15.5 ± 0.3 and 15.5 ± 0.2; polychaetes: − 15.0 ± 1.1 and 7.1 ± 1.6), and intermediated values in Vancouver Island mysids (i.e., − 16.8 ± 3.8 and 11.1 ± 1.0). Based on these data, and on the paradigm that successfully lactating gray whales should have assimilated high proportion of Arctic prey during pregnancy, we expected all female epidermal δ^13^C and δ^15^N values to reflect those of Bering Sea amphipods. Contrary to the postulated hypothesis, diet reconstructions indicated intra and inter-individual differences, among and within years and months of sampling. Only 4 females out of 25 were predicted to have fed in the primary feeding ground of the Bering Sea, while the others appeared to have favored mysids from Vancouver Island (n = 15), or to have integrated prey in both the Bering Sea and Vancouver Island (n = 5). Our results suggest that each sampled mother had a unique feeding strategy, which, consequently, led to variation in the carbon and nitrogen isotope ratios of placental blood and maternal milk assimilated in the epidermis of the respective calves. Previous studies reported behavioral plasticity in feeding eastern gray whales, particularly for individuals that are part of the Pacific Coast Feeding Group^13,15,23^. Nevertheless, we report feeding patterns that differ significantly among almost all individuals, thus it does not seem plausible, although not impossible, that we collected by coincidence only organisms that were part of that specific subgroup.

Generally, our outcome does not support the classical notions related to the eastern gray whale feeding ecology and reproduction. Therefore, the three assumptions of our study are rejected. Particularly, the first two were based on the paradigm that eastern gray whale reproductive success vary among years mainly due to changes in the environmental conditions of the Bering and Chukchi Seas, and because of alterations in the presence and abundance of benthic amphipods^3,4,7,11,45,72^. Consequently, we expected that fluctuations in calf recruitment could be predicted by higher or lower isotopic contributions of Bering Sea amphipods to females’ epidermis. This assumption was only partially confirmed by our data. Females and calf epidermis were collected during years where mother-calf pairs observed in Ojo de Liebre Lagoon differed in their numbers. Particularly, 2011 was a “good” year (> 2000 couples, unpublished data, Biosfera Reserve “El Vizcaino”), while 2018 and 2019 were “bad” ones (1000 or less couples, unpublished data, Biosfera Reserve “El Vizcaino”). Females collected in 2011 integrated in their epidermis similar isotope ratios, which could possibly reflect comparable diets. Mixing models, however, predicted that those animals did not feed exclusively in the Bering Sea, but also near Vancouver Island. Epidermis δ^13^C and δ^15^N values of females collected in 2018 and 2019 were different from those of 2011and varied on an inter-individual basis. Mixing model outcomes reflected those differences, indicating that only few mothers from 2019 fed exclusively upon Bering Sea prey (Fig. 3), while most specimens preferred prey from Vancouver Island. Our results appear puzzling, and future studies should aim to give insights on the feeding dynamics of the eastern gray whale by studying a larger number of individuals sampled during both “good” and “bad” years.

In this study, prey samples were collected in feeding areas a decade earlier than epidermis from 2011, and 16 and 17 years earlier than epidermis from 2018 and 2019. Multiple factors can produce temporal changes in marine organism isotope ratios. The accumulation of carbon and nitrogen stable isotopes along food webs is indeed influenced by changes in the dynamics that occur at the base of each marine trophic system. δ^13^C values can vary due to phytoplankton geometry, size and growth rate^73^, and to seasonal differences in nutrient availability^74^ and sea surface temperature^75^. Moreover, the decrease of differential penetration into oceans of atmospheric CO_2_ enriched in ^12^C, the so-called Suess effect^76^, can influence the distribution of oceanic δ^13^C values leading to a constant decrease in δ^13^C values of plankton with time. On the other hand, variations of δ^15^N values may indicate not only changes in diet composition, as well food-webs reorganizations (for example, the trophic level of prey sources changes with time^77^) or changes in the baseline nitrogen stable isotope ratios^78^. The study of δ^13^C and δ^15^N values recorded in the baleen plates of 37 bowhead whales allowed the investigation of the variation of primary production in the Bering Sea from 1944 to 1997^74^. Precisely, it was documented an average decrease of ~ 2.7‰ in δ^13^C values since 1966^74^, and, successively, and average decrease of 1.3‰ in δ^15^N values since 1952^79^, which were suggested to be related to a decline in the primary productivity of the Bering Sea ecosystem. Concerning the possibility that the Suess-effect could be as well linked to the observed decline in δ^13^C values^80^, Schell (2001)^81^ argued that anthropogenic influxes are much smaller in higher than in lower latitudes, particularly in the North Pacific, because of deep winter mixing and the lack of time for equilibration. The prediction of a decadal increase of δ^13^C values from − 0.1 to 0.2‰ due to the Suess-effect^82^ appears to sustain the hypothesis of a minimization of the anthropogenic effects in the Bering Sea region. Both δ^13^C and δ^15^N values in different species of zooplankton, squids and fish from the Bering Sea and in the Gulf of Alaska are reported to vary interannually, and their range was calculated to be of 0.5–2.0‰ for δ^13^C and of 0.5–2.5‰ for δ^15^N^83^. Furthermore, in the California Current ecosystem, that extends up to Vancouver Island and is located along the migratory route of the eastern gray whale, environmental perturbations like strong El Niño events are known to result in an enrichment in ^15^N at the base of food webs in the California Current zooplankton^84^, quantified to be of ~ 2‰ for different amino acids of different zooplankton groups^85^. Based on this information and giving the lack of data for gray whale prey stable isotope ratios from multiple years, we cannot exclude the possibility that the stable isotope ratios of invertebrates changed between 2002 and 2019. Despite these considerations, we still consider our results valuable, because neither prey or whales’ epidermis were sampled during years with anomalous environmental conditions. Furthermore, the calculated standard error associated with the isotope ratios of Bering Sea amphipods and Vancouver Island mysids was relatively large (i.e., amphipods: ± 1.0‰ for δ^13^C and δ^15^N; mysids: ± 3.8‰ for δ^13^C and ± 1.0‰ for δ^15^N). Consequently, the error margin of our Bayesian mixing models estimates lies within the uncertainty associated with variations in prey isotopic compositions reported above for the Bering Sea and the Gulf of Alaska^83^. Generally, our results indicate that gray whale feeding ecology is not exclusively determined by the consumption of the benthonic amphipods ampeliscid (*Ampelisca macrocephala*), as it was previously assumed^4,8^. Given the implication of our findings, it is critical to determine δ^13^C and δ^15^N values in gray whale prey during multiple years and environmental conditions. This would allow a more precise estimate of whales’ feeding ecology even when sampling of predator and prey do not match temporally, which is often the case not only for the gray whale, but for any other mysticete species.

**Figure 6:**
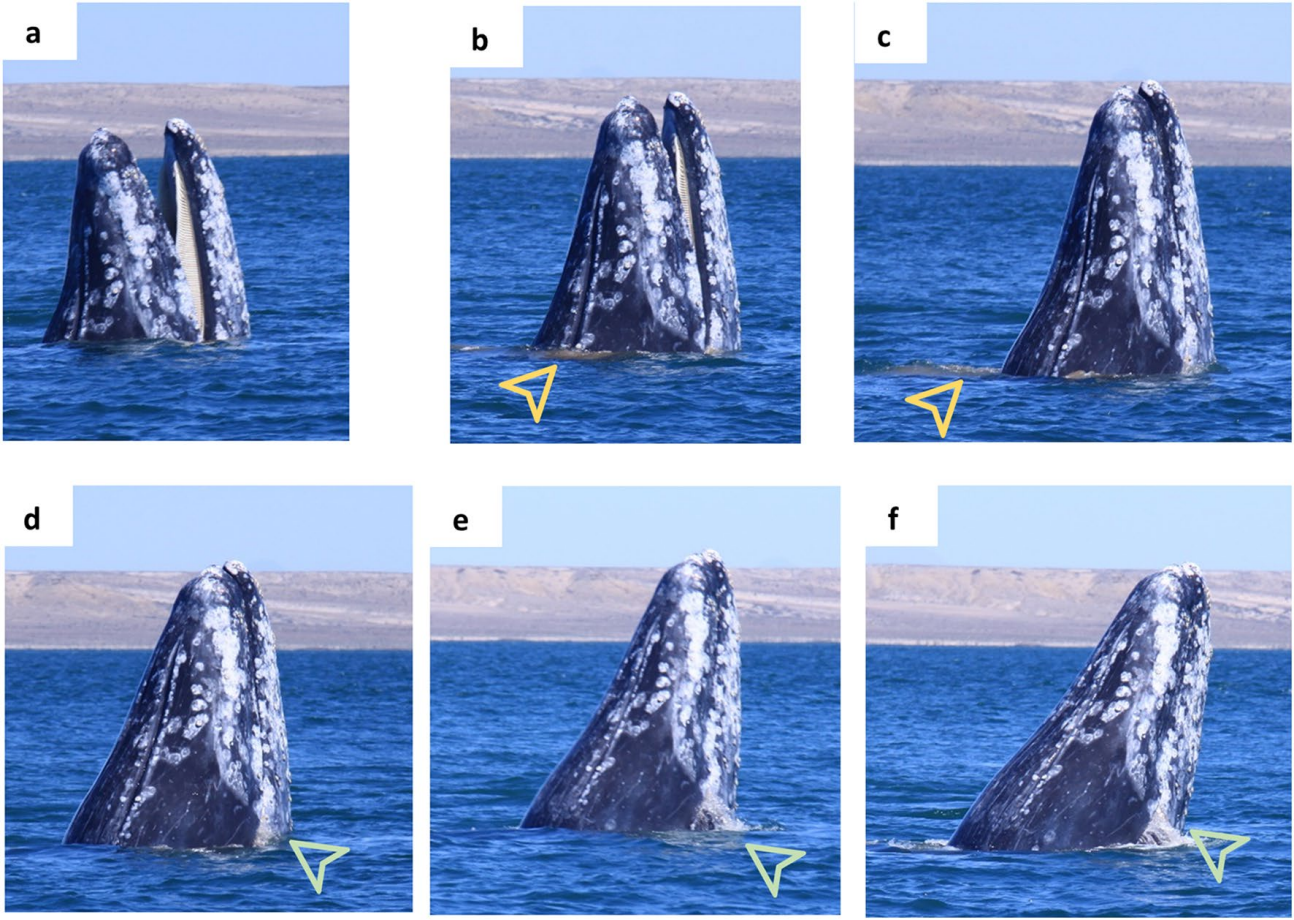
Photographic evidence of a gray whale feeding in Ojo de Liebre Lagoon. The organism emerged with open mouth (**a**,**b**), to then close it (**c**) and expel water (from **d** to **f**, green arrow). The animal then tilted back (**e**,**f**), most probably to better swallow the food. Sediment particles were visible around the whale body after its emersion (**b**,**c**, yellow arrow).

Previous visual observations and isotopic evidence^12,86^ indicated the occurrence of foraging activities in the calving areas. Here, models showed that the probability of contribution of Ojo de Liebre Lagoon prey increased with time (i.e., from SC to SB), independent of year of sampling (Fig. 5). This, together with *in-situ* observations (Fig. 6), suggests a constant use of the breeding lagoon as a feeding ground. Based on results of fatty acid analysis, Caraveo et al*.*^26^ proposed that specific requirements associated with gray whale reproduction could be fulfilled only by feeding in the southern Mexican breeding lagoons and adjacent coasts. High levels of fatty acids omega-6 were found in active tissues^26^, while omega-3 appeared to dominate gray whale blubber^87^. Because mammals cannot synthetize omega-3 and 6 de-novo, and because Arctic food webs are rich in omega-3 fatty acids^88^, while omega-6 availability increases at lower latitudes^89^, these authors^26^ speculated that fatty acids found in whale active tissues could have been assimilated from prey consumed in the breeding lagoons. Our models’ predictions indicate that lactating females’ epidermal δ^13^C and δ^15^N values are determined principally by the intake of prey during gestation, in this case of invertebrates from the Bering Sea and Vancouver Island (Fig. 3a). Conversely, the stable isotope ratios of estimated placental blood (Fig. 3c) and estimated maternal milk (Fig. 3d) appeared to be determined by a combined contribution of energy stored in maternal blubber (i.e., prey from northern feeding grounds) and prey assimilated in the breeding lagoon. This assumption appears to be confirmed by the estimates of source contribution to the sampled-resampled mother-calf pair (i.e., January to March 2019). There, polychaetes from Ojo de Liebre Lagoon contributed similarly to all tissues, following patterns that increased with time (i.e., from mother’s SC to SB, from estimated placental blood to estimated maternal milk) (Fig. 4).

In addition to general constant contribution of Ojo de Liebre Lagoon prey to all organisms, our model outcome highlighted a relation between the kind of organisms consumed in the feeding and in the calving grounds. When *a-priori* models indicated that Ojo de Liebre amphipods had higher probabilities of contribution to female epidermis than polychaetes, *a-posteriori* estimates suggested that Bering Sea amphipods were preferred in the northern feeding grounds, too. On the other hand, when *a-priori* estimates indicated that Ojo de Liebre polychaetes were more exploited than amphipods, *a-posteriori* models predicted a preference for Vancouver Island mysids. The interpretation of these results is complicated by the lack of information on gray whales’ feeding habits in the breeding lagoons. Here, we did not analyze Ojo de Liebre Lagoon prey abundance and distribution, consequently, it is not clear why females should have selected one over the other food item. It is possible that prey availability and abundance vary among and within years, as already reported for amphipods and mysids in the waters around Vancouver Island^15^. Benthic fauna distribution in Ojo de Liebre Lagoon is known to differ on a sediment stability gradient, due to physical changes of the environment and because of biological interactions between organisms^90^. In this study, amphipods and polychaetas were found in seagrass mats and sand, respectively. Among the three study years, seagrass beds reduced in density and distribution in Ojo de Liebre Lagoon (Javier Caraveo-Patiño, personal communication). This could have determined a higher abundance of amphipods in 2011 than in 2018 and 2019, which could therefore explain why 2011 females assimilated only amphipods, despite their ability to implement different feeding strategies, as demonstrated by the intake of both amphipods and mysids in the northern feeding grounds. Another possibility is that some individuals could perform only certain kind of feeding behaviors and, consequently, might not be able to choose between prey items. There exist evidence that gray whale calves learn from their mothers where and how to feed, thus the use of foraging grounds (and consequent prey) is apparently influenced by internal recruitment^22^. If this is true, it is possible that not all gray whales forage in different areas and upon distinct prey. In February 2018, for example, the isotopic composition of female epidermis suggested assimilation of polychaetes, while in January 2019 some integrated only amphipods and others only polychaetes. Given the implications of these assumptions, future studies should focus on the investigation of food web dynamics in the breeding lagoons, to better understand the trophic connectivity that exists between whales and prey items.

Our results stress the necessity to expand investigation efforts all along the eastern gray whale distributional range. The potentially high levels of feeding plasticity highlighted in this study requires further investigations, with a specific focus on the analysis of the isotopic composition of all gray whale possible prey items. As it is already suggested for other baleen whale species, as the humpback^91^, fin^92,93^ and blue^29,93^ whale, our results suggest that gray whale could limit fasting to certain periods. If this is true, other sources than those examined here could contribute importantly to the reproductive success of this species. From 2014 to 2016, for example, an unprecedent marine heatwave (known as “the Blob”) determined short and long-term biological effects from California to Alaska. Precisely, phytoplankton biomass reduced, zooplankton communities got restructured in favor of species poorer in nutrient qualities, resulting in mass mortality of top-predators as fish, seabirds, and marine mammals^94^. Other baleen whales, as the fin and the humpback whales, died in unusually high numbers around Kodiak Island and the western Gulf of Alaska^95^, however the gray whale did not appear to be affected directly by the marine heat wave. In view of our results, however, we cannot discard the possibility that gray whales fed on those new low-caloric prey items for some time, and that this, on the long run, did not affect the population reproductive output, and contributed, instead, to the low number of calves recruited in 2018 and 2019^7^. This is relevant, because the total number of eastern gray whales is expected to decrease and increase cyclically due to short-term anomalous events^96^. Therefore, year-to-year comparison of the changes among female epidermis isotope ratios might detect which feeding habits animals are using during seasons of both high and low reproductive success. Specifically, we suggest continuing to study gray whale feeding ecology by collecting skin biopsies in the breeding lagoons, where animals concentrate in high numbers and environmental conditions are more favorable to sampling than those of northern feeding grounds.

Finally, results of this study should be considered in the design and implementation of management and conservation plans of the endangered western gray whale population. Up to date, low priority was given to all those threats that can determine the degradation and elimination of coastal habitats that are critical for the species life history, mainly because there is no evidence of the dependency of the species to subtropical lagoons as calving and nursery areas. However, 48% of the remaining ~ 300 individuals is estimated to move between the western feeding areas and the eastern reproduction grounds^97^, and that all gray whales need the same kind of habitats to ensure their reproductive output. Based on the findings presented here, it is plausible that if the western gray whale reproduces in the Mexican breeding grounds, it feeds there too. Consequently, it should be a priority to investigate the feeding ecology of both gray whale populations, to understand which nutrients can satisfy their physiological needs, where ideal prey can be found, and how environmental and anthropogenic threats can affect their abundance and distribution.

## Supplementary Information

Supplementary Information.
